# Enhanced CO_2_ Adsorption on Nitrogen-Doped Carbon Materials by Salt and Base Co-Activation Method

**DOI:** 10.3390/ma12081207

**Published:** 2019-04-12

**Authors:** Ruiping Wei, Xingchao Dai, Feng Shi

**Affiliations:** 1 State Key Laboratory for Oxo Synthesis and Selective Oxidation, Lanzhou Institute of Chemical Physics, Chinese Academy of Sciences, No.18, Tianshui Middle Road, Lanzhou 730000, China; wei828111@163.com (R.W.); daixingchao@licp.cas.cn (X.D.); 2 University of Chinese Academy of Sciences, No. 19A, Yuquan Road, Beijing 100049, China

**Keywords:** nitrogen-doped carbon materials, carbon dioxide adsorption, salt and base, co-activation method

## Abstract

Nitrogen-doped carbon materials with enhanced CO_2_ adsorption were prepared by the salt and base co-activation method. First, resorcinol-formaldehyde resin was synthesized with a certain salt as an additive and used as a precursor. Next, the resulting precursor was mixed with KOH and subsequently carbonized under ammonia flow to finally obtain the nitrogen-doped carbon materials. A series of samples, with and without the addition of different salts, were prepared, characterized by XRD (X-ray powder diffraction), elemental analysis, BET (N_2_-adsorption-desorption analysis), XPS (X-ray photoelectron spectroscopy) and SEM (Scanning electron microscopy) and tested for CO_2_ adsorption. The results showed that the salt and base co-activation method has a remarkable enhancing effect on the CO_2_ capture capacity. The combination of KCl and KOH was proved to be the best combination, and 167.15 mg CO_2_ could be adsorbed with 1 g nitrogen-doped carbon at 30 °C under 1 atm pressure. The materials characterizations revealed that the introduction of the base and salt could greatly increase the content of doped nitrogen, the surface area and the amount of formed micropore, which led to enhanced CO_2_ absorption of the carbon materials.

## 1. Introduction

Terrible scenarios of global warming are attributed to the emission of built-up greenhouse gases. Among these greenhouse gases, carbon dioxide (CO_2_), released by the combustion of fuels and from certain industrial and resource extraction processes, is one of the main components. Thus, there are many concerns about reducing carbon dioxide in greenhouse gases. As a result, extensive research efforts have been undertaken to develop feasible materials for CO_2_ capture [1]. Carbon dioxide adsorption especially by porous materials has become a hot research topic because these materials possess many advantages such as low energy requirements, quick and convenient processes of adsorption and desorption compared with chemical absorption [2]. In this context, many porous materials including zeolites [3,4,5], other inorganic molecular sieves [6,7,8,9,10,11,12,13], metal-organic frameworks [14,15,16,17,18,19] and carbon-based materials [20,21,22,23,24] have been investigated.

Among them, carbon-based materials are widely accepted as a promising candidate for CO_2_ adsorption due to their chemical inertness, low cost, high surface area and tunable pore structures. The porous structure and high surface area of carbon materials allow the introduction of several functional groups on the surface to increase the capacity of CO_2_ adsorption. Various carbon-based materials including metal-carbon composites [25], biowaste derived carbons [26,27,28,29] and nitrogen-doped carbons (NC) [30,31,32,33,34,35,36,37,38,39,40,41] have been applied in CO_2_ capture. Among them, nitrogen doped carbon materials have been reported to exhibit an excellent CO_2_ capture capacity and high adsorption selectivity. The incorporation of nitrogen in carbon materials can greatly improve their CO_2_ capture capacity by providing basic adsorption sites. In fact, besides nitrogen-doping, the CO_2_ adsorption of carbon material could also be remarkably enhanced by base activation [42,43]. For example, nitrogen-free microporous materials [44,45,46,47] prepared by alkali etching have been demonstrated to be highly efficient in CO_2_ adsorption. It is noteworthy that alkali etching usually led to the formation of a small amount of micropores and, in other words, the pore structure was changed. Thus, it brings a debate on the exact role of doped nitrogen and pore properties for CO_2_ adsorption. Recently, it has been reported that the pore structure has a determining effect on CO_2_ adsorption at lower temperature and lower pressure, while doped nitrogen plays an important role at higher temperature and higher pressure [48,49,50]. Therefore, it will be highly desirable to develop a porous carbon material enriched in nitrogen and dominated by micropores.

Based on the above discussions, here, we presented nitrogen-doped carbon materials with high CO_2_ capture capacity, which were prepared by the salt and base co-activation method with resorcinol-formaldehyde resin as a precursor. The experimental results showed that the salt and base co-activation method could greatly improve the CO_2_ capture capacity of nitrogen-doped carbon material. The characterization analysis revealed an obvious increase of the doped nitrogen content and the amount of the micropores formed in the carbon material prepared by the salt and base co-activation method, which might be the reason for the enhancement of CO_2_ adsorption. Therefore, a conclusion could be drawn that CO_2_ adsorption was determined by both micropores and the doped nitrogen.

## 2. Materials and Methods

### 2.1. Materials Preparation

Precursors of carbon materials were synthesized by a low temperature hydro-thermal method according to the reported references [51,52,53]. The precursor applied was synthesized as following: Typically, resorcinol (R, 2.20 g, 20 mmol), formaldehyde (F, 3.25 g, 40 mmol, 37 wt % aqueous solution) and 9 mL deionized water were added into a 100 mL Teflon® autoclave. Subsequently, 21.2 mg Na_2_CO_3_ (1 mol % relative to resorcinol) and 0.25–1.25 g salts (KCl, KNO_3_, NaCl, NaNO_3_, Na_2_SO_4_) were added into the autoclave. The mixture was stirred for 1 h at room temperature, and then the autoclave was sealed and kept at 80 °C for 24 h and cooled it down to room temperature to provide an R-F resin (R: resorcinol and F: formaldehyde). The wet resin was put into a round-bottom flask and dried at 130 °C in vacuum condition for 3 h and used as the carbon precursor. Precursor without salt additive was prepared through the same process. Next, the synthesized precursors were mixed mechanically with KOH (0.4–2.0 g) and then carbonized at 400–700 °C (a heating rate of 10 °C min^−1^) for 3 h under ammonia flow (20 mL min^−1^). The resulting carbon materials were ultrasonically washed with deionized water (about 300 mL) until pH ≈ 7.0 and then dried at 80 °C for 6 h to provide the final sample.

### 2.2. CO_2_ Adsorption Measurements

CO_2_ adsorption of the carbon materials was measured using a Mettler-Toledo SDTA851 thermogravimetric analyzer according to the reported references [54,55,56]. In detail, firstly, 10 mg of sample was placed in a porcelain crucible with the volume of 0.1 mL. When the temperature reached 30 °C, the program was started with carbon dioxide (99.9%) as the reaction gas at a flow of 60 mL min^−1^ under 1 atm pressure, and held at that temperature for 50 min. After the completion of the adsorption, the mass of samples after CO_2_ adsorption was recorded as m_1_. Subsequently, the reaction gas was switched to nitrogen (99.9%) with the same flow rate, and at the same time the temperature was increased to 200 °C at a rate of 10 °C min^−1^ and held for 30 min to ensure the complete removal of CO_2_ that samples’ adsorbed. After that, the mass of samples was recorded as m_0_, which is used as the true mass of the sample. CO_2_ adsorption capacity could be calculated by m_1_ and m_0_. 

### 2.3. Characterization Techniques

X-ray powder diffraction (XRD) was performed on a Rigaku D/max-2400 X-ray diffractometer (Rigaku, Tokyo, Japan) with Ni-filtered Cu Kα radiation at 40 kV and 100 mA. The XRD patterns were scanned in the 2θ range of 10–80°.

Elemental analysis (C, N, H and O) of the samples was carried out on a Vario EL microanalyzer (Elementar, Hanau, Germany).

X-ray photoelectron spectroscopy (XPS) was performed by using a Thermo Scientific ESCALAB 250 instrument (Thermo Fisher Scientific, Waltham, MA, USA) with a dual Mg/Al anode X-ray source, a hemispherical capacitor analyser and a 5 keV Ar^+^ ion-gun. All of the spectra were recorded using non-monochromatic Mg Kα (hν = 1253.6 eV) radiation. 

The specific surface area (S_BET_) was calculated using the Brunauer–Emmett–Teller (BET) equation with a relative pressure of 0.05–0.30. The total pore volume (V_Total_) was obtained at the maximum incremental volume point. Micropore volume was determined from the Dubinin–Radushkevic equation. Mesoporous volume was determined by the subtraction of micropore volume from the total pore volume. Fraction of micropore volume = (micropore volume/total pore volume) * 100. The micropore size distribution was calculated by the Harvath–Kawazoe (H–K) equation based on the N_2_/77 K adsorption data.

SEM was performed with a JEOL JSM-6701F (JEOL, Tokyo, Japan) equipped with a cold FEG (Field Emission Gun). 

## 3. Results and Discussion

### 3.1. CO_2_ Adsorption Performance Test

**Figure 1:**
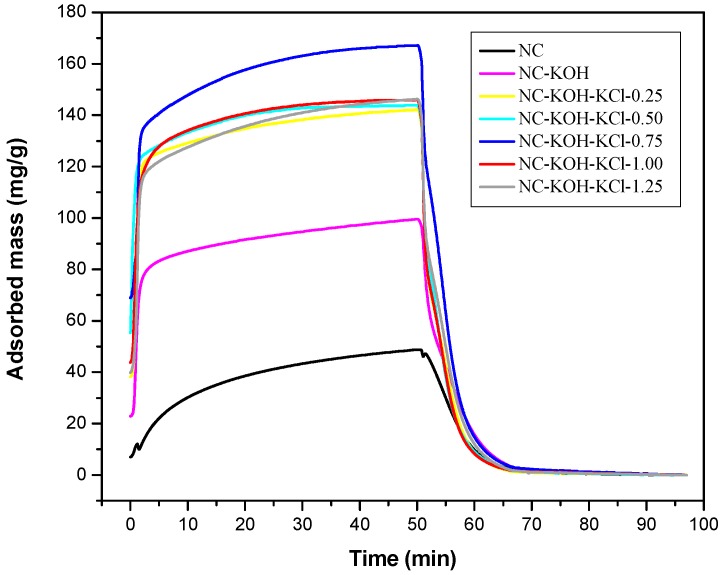
TG curves measured CO_2_ adsorption and desorption of samples activated by different amount of KCl and 1.2 g KOH per 2.20 g resorcinol.

Figure 1 showed the TG curves of CO_2_ adsorption and desorption of these samples activated with 1.2 g KOH and different amount of KCl from 0 to 1.25 g with an interval of 0.25g per 2.20 g resorcinol. The CO_2_ adsorption-desorption behavior was measured at 30 °C under 1 atm. On the basis of the amount of KCl added, these samples were denoted as NC-KOH, NC-KOH-KCl-0.25, NC-KOH-KCl-0.50, NC-KOH-KCl-0.75, NC-KOH-KCl-1.00 and NC-KOH-KCl-1.25. The unactivated sample was denoted as NC. All these samples were carbonized at 600 °C for 3 h under ammonia flow (20 mL min^−1^). It can be seen from the Figure 1, all the samples adsorbed CO_2_ rapidly at the beginning, then continued with a slower rate and reached an equilibrium in 50 min. During the desorption process, the adsorbed CO_2_ is removed rapidly and the mass of samples gradually decreased until a constant value was reached at 200 °C. Figure 1 showed that NC sample had the lowest CO_2_ adsorption capacity and a higher CO_2_ adsorption capacity was observed in the case of NC-KOH sample, which suggested that the introduction of the base in the carbonization process has a positive effect on the increase of CO_2_ adsorption capacity. Similar effect could be also observed by adding the salt in the R-F resin synthesis. Among the tested samples, the samples activated by base and salt exhibited best ability in the CO_2_ adsorption, which could be attributed to the synergistic effect of base and salt pretreatment. However, there is no a linear correlation between the CO_2_ adsorption capacity of the sample and the amount of the salt added. The CO_2_ adsorption capacity of the sample firstly increased then declined, and the maximum (167.15 mg/g) was observed when the sample was activated with 0.75 g KCl and 1.2 g KOH.

**Figure 2:**
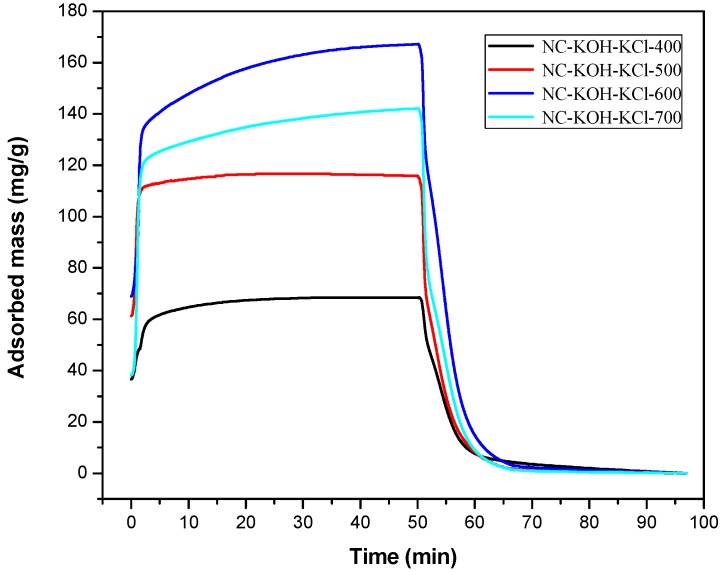
TG curves measured CO_2_ adsorption and desorption of samples carbonized at different temperatures.

After optimizing the amount of KCl, the carbonization temperature of the NC-KOH-KCl-0.75 sample was further optimized in the range of 400–700 °C and the results were shown in the Figure 2. With the increase of the carbonization temperature from 400–600 °C, the CO_2_ adsorption capacity of the sample was gradually enhanced, but a drop was observed when the temperature reached at 700 °C. The best CO_2_ adsorption performance was obtained when the NC-KOH-KCl-0.75 sample was carbonized at 600 °C.

**Figure 3:**
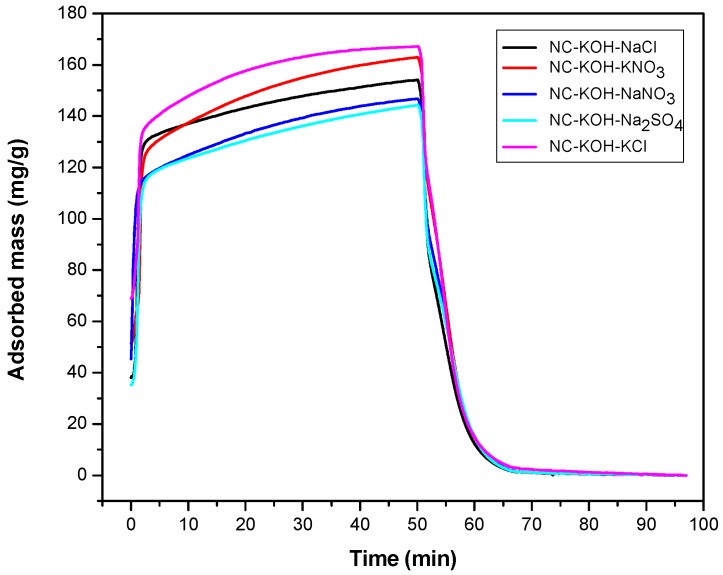
TG curves measured CO_2_ adsorption and desorption of samples activated by different salts.

Following the above results, the effect of the salt kind was investigated (Figure 3). A series of different salts such as KNO_3_, NaNO_3_, KCl, NaCl and Na_2_SO_4_ were added in the R-F resin synthesis process with the optimized amount of 0.75 g and all the samples were carbonized at 600 °C. The results showed that the kind of the salt added has a great effect on the CO_2_ adsorption capacity of the sample. The highest CO_2_ adsorption capacity was obtained when the NC-KOH-KCl sample was used, and 167.15 mg CO_2_ could be adsorbed with 1 g NC-KOH-KCl sample. Slight or much lower values were observed when the other salts such as KNO_3_, NaNO_3_, NaCl and Na_2_SO_4_ were used. Among all the samples tested, the CO_2_ adsorption capacity of the sample activated by Na_2_SO_4_ was the lowest.

**Figure 4:**
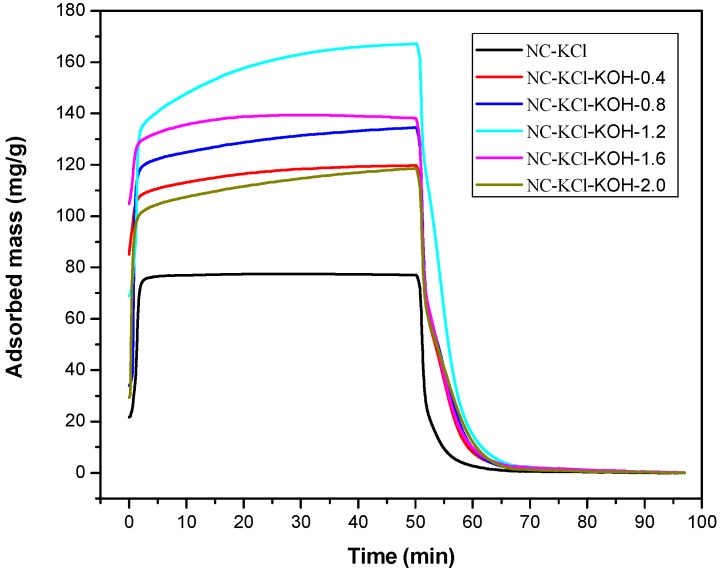
TG curves measured CO_2_ adsorption and desorption of samples activated by different amount of KOH and 0.75 g KCl per 2.20 g resorcinol.

Finally, the amount of KOH added in the carbonization process was optimized in the range of 0–2.0 g and a series of samples activated with 0.75 KCl and different amounts of KOH were prepared. The results in the Figure 4 showed that the CO_2_ adsorption capacity of the NC-KCl-KOH-0.4 sample was much higher than that of the sample activated only with KCl, which indicated that the introduction of KOH could greatly increase the CO_2_ adsorption capacity of the sample. Further increasing the amount of KOH led to higher CO_2_ adsorption capacity, but a sudden drop was observed when 1.6 g KOH was added. The decreased tendency could also be observed when further increasing the amount of KOH to 2.0 g.

**Figure 5:**
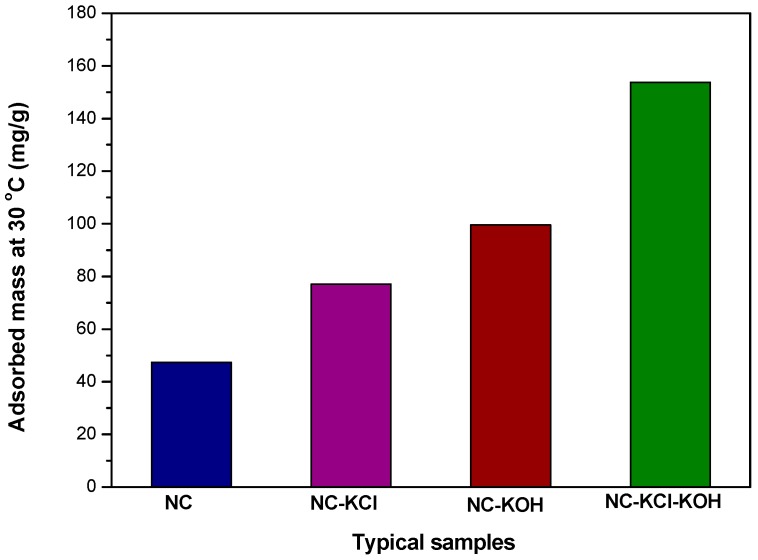
Values of typical samples’CO_2_ adsorption measured by TGA at 30 °C under 1 atm pressure.

In order to better illuminate the effect of salt and base activation on the CO_2_ adsorption capacity of nitrogen-doped carbon materials, four typical samples, e.g., NC, NC-KCl, NC-KOH and NC-KCl-KOH were chosen and further compared (Figure 5). In comparison with NC, NC-KCl and NC-KOH both exhibited better CO_2_ adsorption capacity, which indicated that the base and salt pretreatment both had a promoted effect on the CO_2_ adsorption capacity of the carbon materials, but base is superior to salt by contrast. The highest CO_2_ adsorption capacity was obtained when the NC sample was activated by the combination of base and salt, which could be attributed the synergistic effect of base and salt added in the different steps.

### 3.2. Characterization Results and Discussion

**Figure 6:**
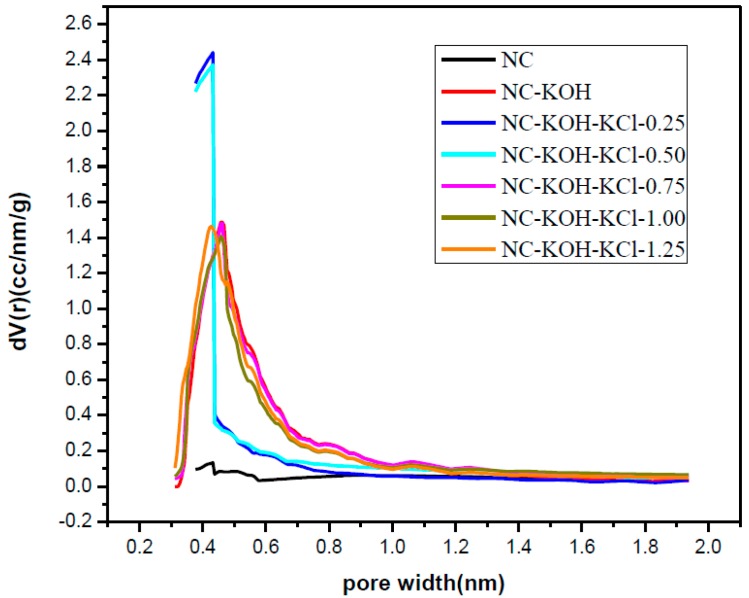
The micropore size distribution of samples activated by different amount of KCl and 1.2 g KOH per 2.20 g resorcinol.

In order to explore the relationship of structure and performance, the prepared samples were characterized by elemental analysis and N_2_-adsorption-desorption analysis, and the results are shown in Table 1 and Table 2, and Figure 6. Obviously, the NC-KOH-KCl-0.75 sample has the highest nitrogen content (12.59 wt %), which implied that the doped nitrogen could promote the CO_2_ adsorption (Table 1). The N_2_-adsorption-desorption analysis revealed that the NC-KOH-KCl-0.75 sample has the largest specific surface areas and highest fraction of micropore volume to total pore volume, which means that the large specific area and more micropores formation might favors the CO_2_ adsorption (Table 2). Thus, the CO_2_ adsorption performance of the carbon material could be affected by the content of the doped nitrogen, the specific area and the amount of micropores.

**Figure 7:**
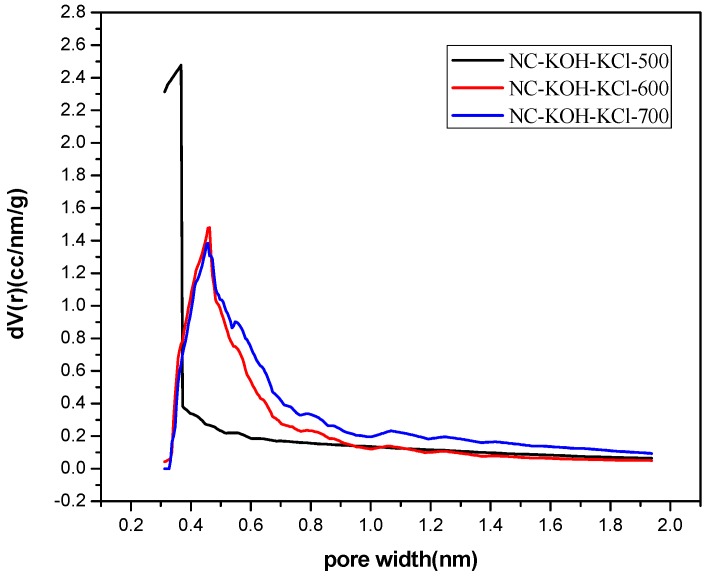
The micropore size distribution of samples carbonized at different temperatures.

Then, the effect of carbonized temperature on the structure was investigated by the elemental analysis and N_2_-adsorption-desorption analysis. It can be seen from the elemental analysis results shown in Table 3 that higher carbonized temperature led to more doped nitrogen in the range of 400–700 °C and the content of doped nitrogen could be increased from 3.5 wt % to 13.08 wt % (Table 3). The NC-KOH-KCl-700 sample has the highest nitrogen content, but its CO_2_ adsorption capacity is not the highest, which means that the CO_2_ adsorption capacity of the carbon material was not determined by only the content of the doped nitrogen. Further, N_2_-adsorption-desorption analysis revealed a good correlation between the micropore volume and the carbonized temperature. In addition, micropore volume enlarged with the increase of the carbonized temperature (Table 4 and Figure 7). The NC-KOH-KCl-600 sample with the best CO_2_ adsorption performance has the highest fraction of micropore volume to total pore volume, which is consistent with the above discussions.

**Figure 8:**
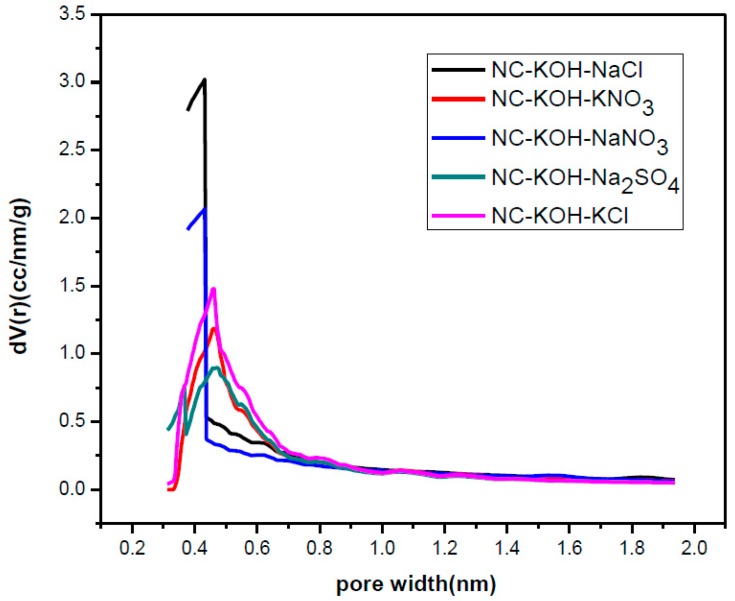
The micropore size distribution of samples activated by different salts.

Next, the samples activated by KOH and different salts were characterized by elemental analysis and N_2_-adsorption-desorption analysis and the results were shown in Table 5 and Table 6 and Figure 8. Obviously, these samples co-activated by base and salt have high nitrogen content and all exceeded 12 wt % (Table 5). Especially, for the samples activated by KNO_3_, NaNO_3_ and Na_2_SO_4_, the nitrogen content above 15 wt % was observed. The N_2_-adsorption-desorption analysis revealed that the kind of the activated salt has a great effect on the pore structure of the carbon material. The samples activated by KCl and NaCl exhibited a specific surface area above 1000 m^2^ g^−1^ while the smaller specific surface area than 1000 m^2^ g^−1^ were observed in the case of other salts (Table 6). Similar phenomena were also observed in the case of total pore volume and micropore volume. It is noteworthy that the NC-KOH-KCl sample exhibited the highest fraction of micropore volume to total pore volume although its micropore volume is not the largest, which suggested that a larger micropore volume did not mean higher CO_2_ adsorption capacity.

**Figure 9:**
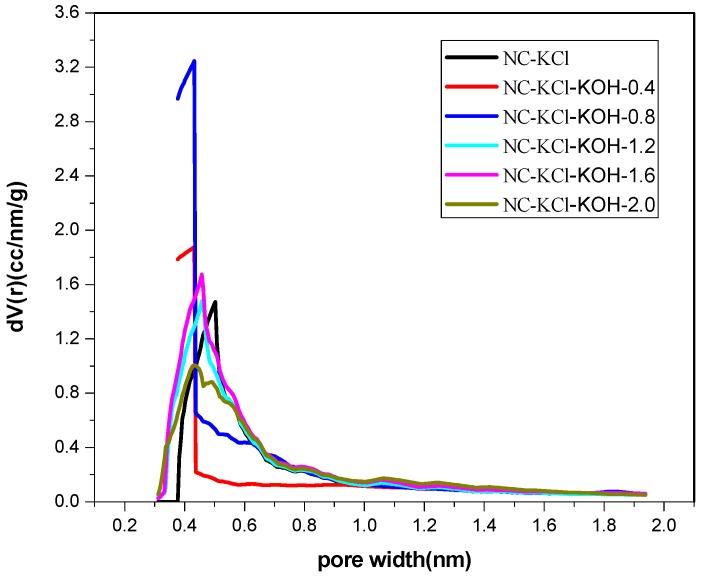
The micropore size distribution of samples activated by different amount of KOH and 0.75 g KCl per 2.20 g resorcinol.

Furthermore, in order to explore the difference in the structure of the samples activated by different amount of KOH, these samples were characterized by elemental analysis and N_2_-adsorption-desorption analysis and the results were shown in Table 7 and Table 8 and Figure 9. The elemental analysis showed that the nitrogen content in the NC-KCl sample was 5.28 wt % and the value could be increased to 11.2 wt % by adding 0.4 g KOH (Table 7), which implied that the introduction of KOH could greatly increase the nitrogen content. The addition of more KOH led to higher nitrogen content, but slight -promotion effect was observed if the amount of KOH exceeded 1.2 g. Besides, the promotion effect of KOH was also observed in the specific area. Apart from the NC-KCl-KOH-0.4 sample (Table 8), all the other samples activated by KOH exhibited a larger specific area than the NC-KCl sample, which suggested that the introduction of KOH could increase the specific area of the carbon material, but a certain amount of KOH was required.

**Figure 10:**
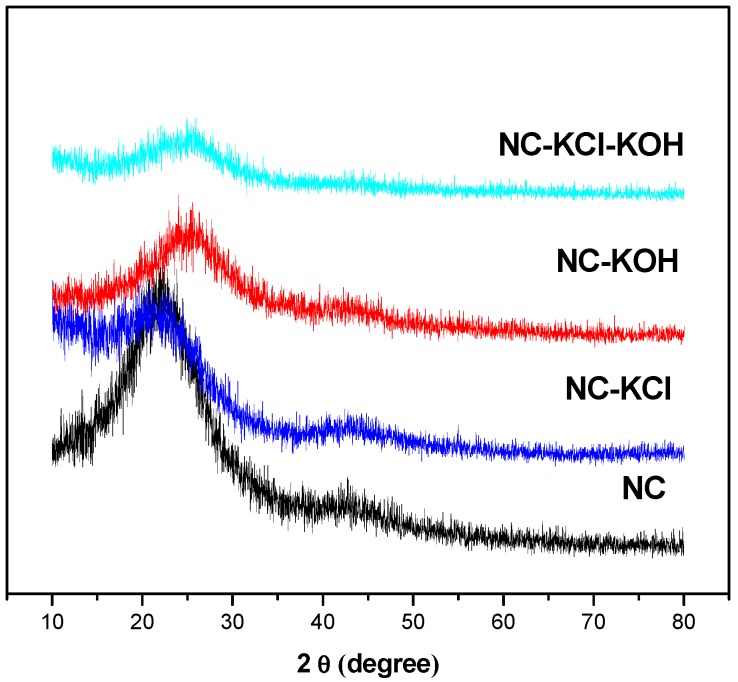
X-ray diffraction patterns of typical samples.

XRD patterns of the sample were shown in the Figure 10 and a typical reflection of amorphous carbon at about 24° was observed in all samples, which could be assigned to hexagonal graphite [47]. Besides, a weak peak appeared at approximately 43° in all the samples but NC-KCl-KOH sample, which could be assigned to rhombohedral graphite [47]. By correlating with the CO_2_ adsorption capacity, it’s not difficult to make a speculation that the formation of rhombohedral graphite might produce adverse effect for the CO_2_ adsorption.

The contents of C, H and N in the sample were determined by elemental analysis, and the content of O was calculated by the subtracting from the content of C, H and N from the total content. The results in Table 9 showed that the activation of the sample by base and salt has a great effect on the content of N. The nitrogen content in the NC sample was 7.78 wt %, and the value could be increased to 11.99 wt % by the KOH activation, which suggested that the introduction of KOH might favors the formation of nitrogen-containing functional groups during ammoxidation process. As is well known, the existence of the doped nitrogen could provide the basic sites to adsorb CO_2_ and higher nitrogen content means more CO_2_ adsorption sites. Therefore, the higher CO_2_ adsorption capacity of the NC-KOH exhibited could be well explained. A similar increase in the nitrogen was observed when the sample was activated by KOH and KCl, which further confirmed the effect of KOH. Considering the enhanced CO_2_ adsorption capacity of NC-KOH and NC-KCl-KOH samples in comparison with NC, a speculation could be made that the high nitrogen content in the sample is good for the CO_2_ adsorption by providing more basic sites. It is worth noting that the nitrogen content of the sample activated by KCl decreased, but its CO_2_ adsorption capacity reversely increased, which implied that the introduction of KCl might increase the CO_2_ adsorption capacity by changing the sample’s pore structures not increasing the nitrogen content.

Then the porosity and BET specific surface area of typical samples were determined by N_2_ adsorption and desorption and the results are shown in Table 10. Obviously, the samples activated by KOH and/or KCl had a larger surface area than the NC sample, and the NC-KCl-KOH sample with the highest CO_2_ adsorption capacity exhibited the largest surface area, which suggested that the large surface area might be favorable the CO_2_ adsorption. A similar phenomenon could also be observed in the pore volume of the sample and the pore volume of the sample could be increased from 0.19 cm^3^ g^−1^ to 0.634 cm^3^ g^−1^ by the co-activation of KOH and KCl. However, the NC-KCl sample showed the biggest pore volume although its CO_2_ adsorption capacity was lower than that of the NC-KOH and NC-KCl-KOH samples. In order to gain more insights on the correlation of the pore volume and the CO_2_ adsorption capacity, the micropore and mesoporous volume as well as the fraction of micropore volume to total pore volume were calculated, respectively. A linear correlation between the CO_2_ adsorption capacity and the fraction of micropore volume to total pore volume could be observed, which suggested that the formation of the micropore should be important for the CO_2_ adsorption.

Based on the above discussions, the CO_2_ adsorption capacity of the sample was determined by the nitrogen content and the pore structure. By contrast, the latter played a more important role. 

**Figure 11:**
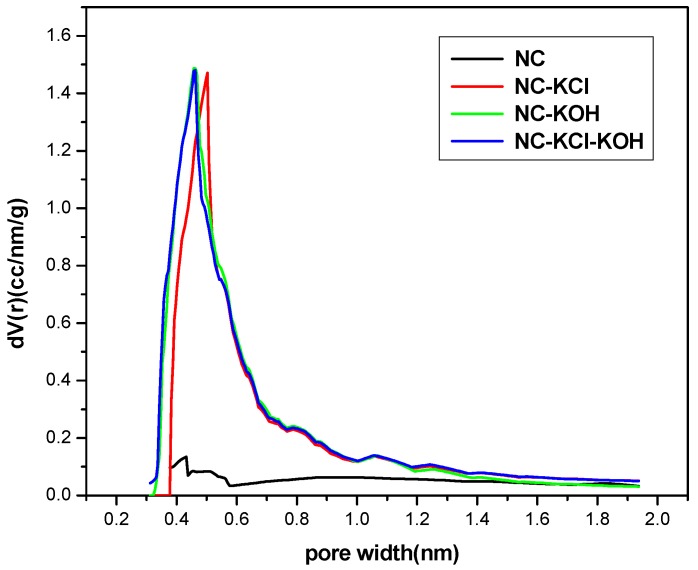
The micropore size distribution of typical samples.

The micropore size distribution of typical samples was characterized by using the Harvath–Kawazoe (H–K) equation based on the N_2_ adsorption and desorption data (Figure 11). Obviously, the micropores in the NC sample were very few, which is consistent with its small micropore volume presented in Table 2. The introduction of KCl in the R-F resin synthesis could promote the formation of more micropores and the micropore size ranged from 0.3–1.9 nm with a peak at 0.50 nm. Similar effect could be also observed when adding KOH in the carbonization process of the material. It’s different from the NC-KCl sample that the NC-KOH sample had more micropores with smaller pore size and the peak value shifted left to 0.46 nm. The NC-KCl-KOH sample exhibited a nearly same micropore structure with the NC-KOH sample. It is noteworthy that the size of most micropores in the three samples activated by base and/or salt is smaller than 0.7 nm, and these pores were reported to support the CO_2_ adsorption [48].

**Figure 12:**
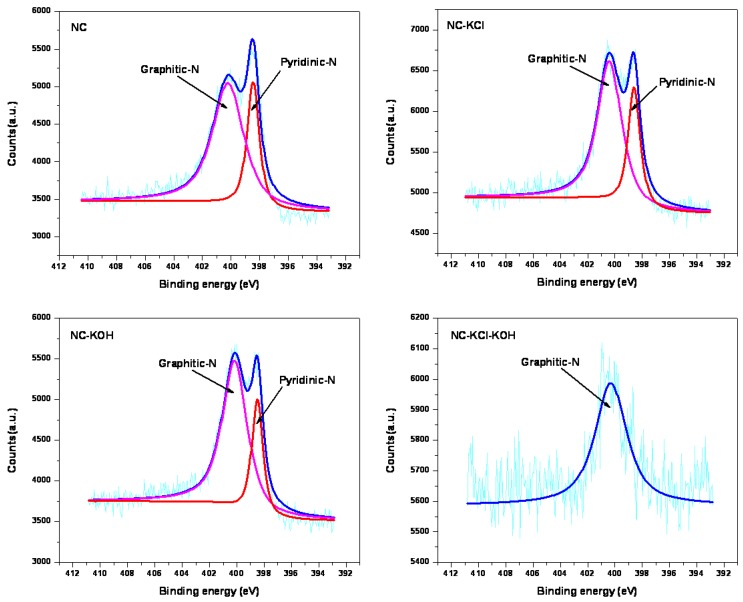
N1s XPS spectra of typical samples.

The nitrogen bonding configurations were further studied by XPS and the N 1s spectra of typical samples are shown in Figure 12. Two signal peaks with binding energy at 398.5 and 400.3 eV were observed in all the samples except for NC-KCl-KOH sample. The peak at 398.5 eV could be assigned to pyridinic nitrogen and the peak at 400.3 eV to graphitic nitrogen [49]. It can be found that the ratio of pyridinic nitrogen to graphitic nitrogen was greatly influenced by the pre-treatment activation by comparing the relative intensity of pyridinic and graphitic nitrogen peaks. The salt and base activation pre-treatment led to a decrease in the ratio of pyridinic nitrogen to graphitic nitrogen. In the case of NC-KCl-KOH sample, only the signal peak corresponding to graphitic nitrogen was observed, which suggested that graphitic nitrogen could behave as effective binding sites for CO_2_. It has been reported that different kinds of nitrogen functional groups have different degrees of effects on materials’ CO_2_ adsorption [32], which is also the reason for that the material’s nitrogen content obtained by element analysis could not match its CO_2_ adsorption performance very well. 

**Figure 13:**
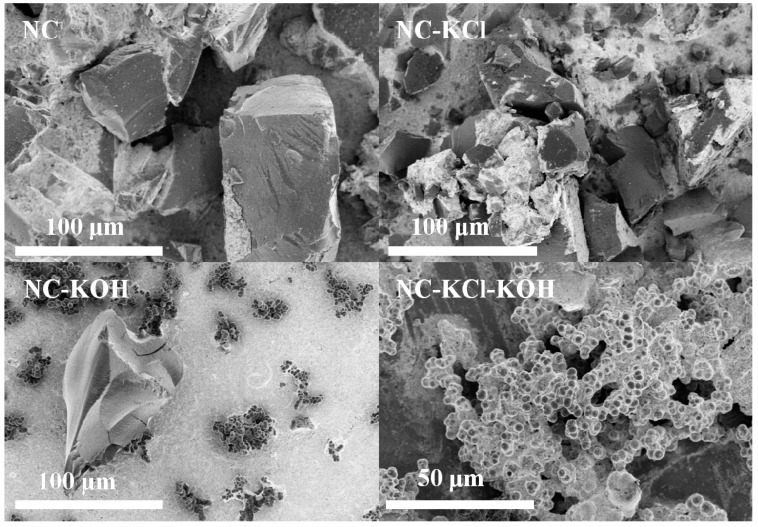
SEM image of typical samples and their precursors.

Finally, the surface morphology of typical samples and their precursors were analyzed by SEM and the results were shown in Figure 13. The morphology of the precursor without KCl activation took on like-lumps feature. When adding 0.75 g KCl in the precursor synthesis, the morphology could be changed to form uniform and close-connected small spheres. However, the surface of the NC and NC-KCl samples both consisted of carbon blocks with different size although the latter contained more carbon blocks with smaller size. Besides irregular carbon blocks, some uniformly small carbon spheres could be also observed when introducing 1.2 g KOH in the carbonization process of the precursor without KCl activation pretreatment, which could be attributed to the etch effect of base. Different from the above case, only uniformly small carbon spheres were obtained when the sample was co-activated by 0.75 KCl and 1.2 g KOH, which suggested a synergistic effect of base and salt activation pretreatment on the regulation of the morphology.

## 4. Conclusions

In this work, a series of nitrogen-doped carbon materials with high CO_2_ capture capacity were prepared by the ammoxidation of resorcinol-formaldehyde resin precursor with the aid of salt and/or base pretreatment activation. An obvious synergistic effect was observed between base and salt and the combination of 0.75 g KCl and 1.2 g KOH was proven to be the best combination. The sample co-activated by KCl and KOH exhibited the best CO_2_ adsorption performance and 1 g typical NC-KCl-KOH sample could adsorb up to 167.15 mg CO_2_. The extensive characterization revealed that the introduction of KCl and KOH could increase the doped nitrogen content, change the nitrogen bonding configurations, enlarge the specific surface area and promote the formation of micropores with the size <0.7 nm. Therefore, the CO_2_ adsorption capacity of the nitrogen doped carbon material should be co-influenced by the amount and type of doped nitrogen and the pore structure. 

## Figures and Tables

**Table 1:** The content of N, C, H and O in samples activated by different amount of KCl and 1.2 g KOH per 2.20 g resorcinol.

Samples	N (wt %)	C (wt %)	H (wt %)	O (wt %)
NC	7.78	76.12	1.91	14.19
NC-KOH	11.99	58.22	2.67	27.12
NC-KOH-KCl-0.25	7.05	69.19	1.76	22.00
NC-KOH-KCl-0.50	8.40	70.09	1.88	19.63
NC-KOH-KCl-0.75	12.59	56.23	2.58	28.60
NC-KOH-KCl-1.00	9.43	68.05	1.88	20.64
NC-KOH-KCl-1.25	8.70	66.12	1.74	23.44

**Table 2:** BET surface area and porosity of samples activated by different amount of KCl and 1.2 g KOH per 2.20 g resorcinol.

Samples	S_BET_ (m^2^ g^−1^) ^1^	V_total_ (cm^3^ g^−1^) ^2^	V_Micro_ (cm^3^ g^−1^) ^3^	V_Meso_ (cm^3^ g^−1^) ^4^	F_Micro_ (%) ^5^
NC	158	0.190	0.055	0.135	29
NC-KOH	1030	0.659	0.401	0.258	61
NC-KOH-KCl-0.25	911	1.013	0.354	0.659	35
NC-KOH-KCl-0.50	900	0.573	0.349	0.224	61
NC-KOH-KCl-0.75	1034	0.634	0.398	0.236	63
NC-KOH-KCl-1.00	858	0.740	0.361	0.379	49
NC-KOH-KCl-1.25	1006	0.615	0.389	0.226	63

^1^ S_BET_ is the specific surface areas determined by the BET method. ^2^ V_Total_ is the total pore volume. ^3^ V_Micro_ is the micropore volume. ^4^ V_Meso_ is the mesoporous volume. ^5^ F_Micro_ is the fraction of micropore volume to total pore volume.

**Table 3:** The content of N, C, H and O in samples carbonized at different temperatures.

Samples	N (wt %)	C (wt %)	H (wt %)	O (wt %)
NC-KOH-KCl-400	3.5	66.00	2.13	28.37
NC-KOH-KCl-500	7.74	69.58	1.84	20.84
NC-KOH-KCl-600	12.59	56.23	2.58	28.60
NC-KOH-KCl-700	13.08	63.95	1.30	21.67

**Table 4:** BET surface area and porosity of samples carbonized at different temperatures.

Samples	S_BET_ (m^2^ g^−1^) ^1^	V_total_ (cm^3^ g^−1^) ^2^	V_Micro_ (cm^3^ g^−1^) ^3^	V_Meso_ (cm^3^ g^−1^) ^4^	F_Micro_ (%) ^5^
NC-KOH-KCl-400	167	0.210	0.009	0.201	4
NC-KOH-KCl-500	959	0.671	0.369	0.302	55
NC-KOH-KCl-600	1034	0.634	0.398	0.236	63
NC-KOH-KCl-700	1300	0.812	0.483	0.329	59

^1^ S_BET_ is the specific surface areas determined by the BET method. ^2^ V_Total_ is the total pore volume. ^3^ V_Micro_ is the micropore volume. ^4^ V_Meso_ is the mesoporous volume. ^5^ F_Micro_ is the fraction of micropore volume to total pore volume.

**Table 5:** The content of N, C, H and O in samples activated by different salts.

Samples	N (wt %)	C (wt %)	H (wt %)	O (wt %)
NC-KOH-NaCl	12.27	59.51	1.53	26.69
NC-KOH-KNO_3_	15.45	56.01	1.51	27.03
NC-KOH-NaNO_3_	15.37	67.18	1.57	15.88
NC-KOH-Na_2_SO_4_	15.22	58.14	1.71	24.93
NC-KOH-KCl	12.59	56.23	2.58	28.60

**Table 6:** BET surface area and porosity of samples activated by different salts.

Samples	S_BET_ (m^2^ g^−1^) ^1^	V_total_ (cm^3^ g^−1^) ^2^	V_Micro_ (cm^3^ g^−1^) ^3^	V_Meso_ (cm^3^ g^−1^) ^4^	F_Micro_ (%) ^5^
NC-KOH-NaCl	1217	0.775	0.466	0.309	60
NC-KOH-KNO_3_	874	0.629	0.327	0.302	52
NC-KOH-NaNO_3_	854	0.620	0.323	0.297	52
NC-KOH-Na_2_SO_4_	926	0.602	0.354	0.248	59
NC-KOH-KCl	1034	0.634	0.398	0.236	63

^1^ S_BET_ is the specific surface areas determined by the BET method. ^2^ V_Total_ is the total pore volume. ^3^ V_Micro_ is the micropore volume. ^4^ V_Meso_ is the mesoporous volume. ^5^ F_Micro_ is the fraction of micropore volume to total pore volume.

**Table 7:** The content of N, C, H and O in samples activated by different amount of KOH and 0.75g KCl per 2.20 g resorcinol.

Samples	N (wt %)	C (wt %)	H (wt %)	O (wt %)
NC-KCl	5.28	65.28	2.51	26.93
NC-KCl-KOH-0.4	11.2	73.27	1.4	14.13
NC-KCl-KOH-0.8	12.54	61.97	1.39	24.1
NC-KCl-KOH-1.2	12.59	56.23	2.58	28.60
NC-KCl-KOH-1.6	12.65	63.23	1.54	22.58
NC-KCl-KOH-2.0	12.96	58.89	1.4	26.75

**Table 8:** BET surface area and porosity of samples activated by different amount of KOH and 0.75g KCl per 2.20 g resorcinol.

Samples	S_BET_ (m^2^ g^−1^) ^1^	V_total_ (cm^3^ g^−1^) ^2^	V_Micro_ (cm^3^ g^−1^) ^3^	V_Meso_ (cm^3^ g^−1^) ^4^	F_Micro_ (%) ^5^
NC-KCl	903	0.686	0.348	0.338	51
NC-KCl-KOH-0.4	702	0.518	0.269	0.249	52
NC-KCl-KOH-0.8	1352	0.758	0.517	0.241	68
NC-KCl-KOH-1.2	1034	0.634	0.398	0.236	63
NC-KCl-KOH-1.6	1159	0.722	0.445	0.277	62
NC-KCl-KOH-2.0	999	0.575	0.377	0.198	66

^1^ S_BET_ is the specific surface areasdetermined by the BET method. ^2^ V_Total_ is the total pore volume. ^3^ V_Micro_ is the micropore volume. ^4^ V_Meso_ is the mesoporous volume. ^5^ F_Micro_ is the fraction of micropore volume to total pore volume.

**Table 9:** The content of N, C, H and O in the typical samples determined by elemental analysis.

Samples	N (wt %)	C (wt %)	H (wt %)	O (wt %)
NC	7.78	76.12	1.91	14.19
NC-KCl	5.28	65.28	2.51	26.93
NC-KOH	11.99	58.22	2.67	27.12
NC-KCl-KOH	12.59	56.23	2.58	28.60

**Table 10:** BET surface area and porosity of typical samples.

Samples	S_BET_ (m^2^ g^−1^) ^1^	V_total_ (cm^3^ g^−1^) ^2^	V_Micro_ (cm^3^ g^−1^) ^3^	V_Meso_ (cm^3^ g^−1^) ^4^	F_Micro_ (%) ^5^
NC	158	0.190	0.055	0.135	29
NC-KCl	903	0.686	0.348	0.338	51
NC-KOH	1030	0.659	0.401	0.258	61
NC-KCl-KOH	1034	0.634	0.398	0.236	63

^1^ S_BET_ is the specific surface areasdetermined by the BET method. ^2^ V_Total_ is the total pore volume. ^3^ V_Micro_ is the micropore volume. ^4^ V_Meso_ is the mesoporous volume. ^5^ F_Micro_ is the fraction of micropore volume to total pore volume.
